# Slowing down light using a dendritic cell cluster metasurface waveguide

**DOI:** 10.1038/srep37856

**Published:** 2016-11-25

**Authors:** Z. H. Fang, H. Chen, F. S. Yang, C. R. Luo, X. P. Zhao

**Affiliations:** 1 Smart Materials Laboratory, Department of Applied Physics, Northwestern Polytechnical University, Xi’an 710129 P.R. China

## Abstract

Slowing down or even stopping light is the first task to realising optical information transmission and storage. Theoretical studies have revealed that metamaterials can slow down or even stop light; however, the difficulty of preparing metamaterials that operate in visible light hinders progress in the research of slowing or stopping light. Metasurfaces provide a new opportunity to make progress in such research. In this paper, we propose a dendritic cell cluster metasurface consisting of dendritic structures. The simulation results show that dendritic structure can realise abnormal reflection and refraction effects. Single- and double-layer dendritic metasurfaces that respond in visible light were prepared by electrochemical deposition. Abnormal Goos-Hänchen (GH) shifts were experimentally obtained. The rainbow trapping effect was observed in a waveguide constructed using the dendritic metasurface sample. The incident white light was separated into seven colours ranging from blue to red light. The measured transmission energy in the waveguide showed that the energy escaping from the waveguide was zero at the resonant frequency of the sample under a certain amount of incident light. The proposed metasurface has a simple preparation process, functions in visible light, and can be readily extended to the infrared band and communication wavelengths.

Making light travel slower rather than faster is possibly the main challenge in optical transmission^1^. Slowing light promotes stronger light–matter interaction, it offers additional control over the spectral bandwidth of this interaction, and it allows us to delay and temporarily store light in all-optical memories^2^. Most of the approaches to obtain slow light rely on the phenomenon of electromagnetically induced transparency (EIT)^3^ in ultracold or warm atomic gases^4^,^5^, plasmas^6^,^7^ and a doped crystal^8^; such systems are excellent for fundamental investigations, but are unsuitable for practical applications because of the harsh experimental conditions.

Metamaterials with negative refractive indexes are ideal materials for use in slowing down light^9^,^10^. Metamaterials enable the engineering of electromagnetic resonances with nearly arbitrary frequencies and spatial symmetries^11^. In 2007, Hess *et al*. demonstrated theoretically that an axially varying heterostructure could be used to efficiently and coherently bring light to a complete standstill, i.e. “rainbow trapped”^12^. However, scientists must develop nanoscale materials to realise the true interception of light^13^,^14^. In 2009, Zhao *et al*. experimentally demonstrated the trapped rainbow in tapered left-handed heterostructures at visible frequencies^14^. However, the internal high losses of metamaterials limit the storage time and interaction length^2^. Moreover, the dispersion effect of metamaterials limits the available bandwidth. Additionally, difficulties in the preparation of metamaterials further limit the development of slow light. In 2011, Capasso and Gaburro respectively suggested two types of metasurface models that could control the direction of the reflection and refraction of light by the wavefront phase discontinuity in interface structure^15^,^16^. These metasurface structures bring new opportunities for the modification of optical transmission realised by metamaterials^17^. Currently, most of the metasurface structures are regular periodic structures, such as the double-pole structure^18^, the H-type structure^19^, and the structure comprised of U-shaped elements^20^,^21^,^22^. Metasurface structures have been demonstrated to produce a response across the spectral range from microwave and infrared wavelengths. In 2015, Alexander reported a visible frequency hyperbolic metasurface that displayed both negative refraction and diffraction-free propagation^23^. However, most of the aforementioned metasurfaces have been fabricated using top-down approaches of lithography or an etching technique; such techniques are not conducive to practical applications because of the high cost and extremely small sample volume sizes.

To address the bottleneck currently posed by the preparation of metasurfaces in visible light, we propose a dendritic cell cluster metasurface (also referred to as a dendritic metasurface) prepared via a bottom-up fabrication method. This method is facile and low cost^24^,^25^. Numerical simulations confirm that anomalous reflection and anomalous refraction effects are obtained from such a metasurface. We prepared single- and double-layer silver dendritic metasurfaces by electrochemical deposition. An abnormal GH shift^26^,^27^ can be realised using the silver dendritic metasurfaces. The waveguide sample based on the dendritic metasurfaces presents a rainbow trapping effect. Our further measurements show that the light power at the outlet of the waveguide is zero when a certain energy of light is launched into the waveguide. This is an important step in optical information transmission and storage technology research, and has broad application prospects and development potential.

As we all known that, the basic units of metamaterials are metal wire and split-ring resonator. The metal wire array can achieve the negative permittivity *ε* and the split-ring resonator array can achieve negative permeability *μ*. We suggested dendritic structure as a mergence of metal wire and split-ring resonator. In 2007, Zhou *et al*. proved that dendritic structure operate in microwave can achieve negative *ε* and *μ*^28^. In 2008, Liu *et al*. confirmed that dendritic structure can achieve negative *ε* and *μ* meanwhile in infrared^29^. The metasurface was proposed in 2011. Yu *et al*. proposed optically thin arrays of metallic antennas, which composed of rod and V-type units, realized the anomalous reflection and refraction phenomena which are in excellent agreement with generalized Snell’s law^15^. Based on the accumulate in the dendritic metamaterials, we suggested the dendritic metasurface. A simulated method which is same to the method in ref. 15 is employed to simulate the dendritic metasurface. The geometrical structure of the dendritic metasurface is shown in the inset of Fig. 1a. For detailed characteristics of each geometrical structure of the silver dendritic cluster, please refer to Supplementary Materials 1. The transmission of the reflected and refracted waves can be observed using the Computer Simulation Technology (CST) programme to simulate the incidence of the plane wave on the metasurface. Both the electrical and the magnetic field distribution on the metasurface are shown. The simulation results suggest that a dendritic structure exhibits abnormal phenomena, such as negative refraction. Three different shapes of dendritic cell metasurface have identical efficacy, as shown in Supplementary Material 1. Each kind of metasurface is composed by a single type of dendritic structure. The simulated results reveal that the transmission curve with a single resonance frequency can achieve using a metasurface model composed by a single type of dendritic structure. However, there is a large number of dendritic units in the real fabricated metasurface sample. It is obviously that we can’t design a model which is exactly the same to the real fabricated metasurface sample. Therefore, we have to carry out the simulation using limited types of dendritic structure, and with hope to get consistent with the experimental results. The results of repeated optimization of many types of dendritic structure reveals that: When three types of dendritic structure randomly arranged to form a dendritic cell cluster, and the number of each type of dendritic structure in a cluster is 3 (Fig. 1a), was employed in simulation, the diversity of different dendritic units in real fabricated sample was represented, in addition, the simulated transmission coefficient curves which is closed to the experimental result was obtained in suitable amount of calculation. Thus, these three dendritic structures were used to represent the real fabricated metasurface sample. The reflected and refracted waves exhibit abnormal transmission, as they are on the same side of the normal as the incident wave (Fig. 1c). By extracting the field distribution data and calculating S = (1/2)Re(E*H) with Matlab, the power flux distribution of the reflected and refracted waves can be acquired, as shown in Fig. 1d, where the arrows indicate the direction. The directions of the power flux of the reflected wave and the refraction wave are identical to the wave vector, i.e. they are all transmitted along the -x-axis. In traditional left-handed materials, the power flux is in an opposite direction to the wave vector, whereas the energy transmits forwards continually. However, for the dendritic metasurface the direction of the power flux is identical to the wave vector for the reflected and refracted waves.

The ***E*** and ***H*** field distributions of the simulation results for the three types of silver dendritic structures show that the transmission of the light wave follows Snell’s general law, i.e. here, the reflective angle is not equal to the incident and has a negative relationship to the incident angle. The relationship between the refracted angle and the incident angle does not follow the normal refractive law, i.e. the negative refraction phenomenon appears when the light wave travels through the silver dendritic structure. Furthermore, the transmission coefficient and phase curves obtained by the simulation indicate that an abrupt change of phase occurs at a resonant wavelength of approximately 614 nm. Therefore, the structure can provide the required phase change for abnormal refraction. It is well known that an array of dendritic unit structures with different shapes and sizes can be prepared using electrochemical techniques^29^, i.e. a “bottom-up” method of chemical preparation. Therefore, the dendritic cell cluster provides a theoretical basis for the “bottom-up” preparation of metasurfaces that operate in visible light.

The preparation method and the properties of the metasurface sample are shown in Fig. 2. Figure 2a shows the preparation process of single- and double-layer samples. We obtained single- and double-layer silver dendritic structures with areas of 13 mm × 10 mm on an Indium Tin Oxides (ITO) conductive glass surface using the electrochemical workstation Autolab. The results from hundreds of experiments indicate that the major factors of the preparation of a sample are the density of the electrolytic solution as well as the sedimentation voltage and time. Several resonances at different frequencies or a single resonance at a certain frequency can be achieved through the adjustment of these factors. In this paper, we recommend the use of a silver dendritic metasurface with a single resonance frequency of either red light or green light. Because the sizes of samples in fabrication cannot be made to match the simulations, i.e. some variance from the simulation is found. We suggest that three types of dendritic cell are possible, as shown in Fig. 1. The simulated transmission curve is close to the experimental result. More computation is necessary when the number of units is larger. Thus, we suggest the use of a cluster composed of nine dendritic units of three types, which may well represent the silver dendritic metasurface with a single resonance in this paper.

Figure 2b and c are scanning electron micrographs of single- and double-layer silver dendritic metasurfaces. The single-layer dendritic unit is uniformly distributed onto the substrate surface. The diameter of the dendritic unit is approximately 200–300 nm. The photograph of the double-layer silver dendritic structure is less clear than that of a single-layer silver dendritic structure because of the middle dielectric medium layer. The diameter of the second layer of the silver dendritic unit is 450 nm. The geometry of the structures is close to that expected from the design and simulations. Figure 2d and e show the transmission spectra of the samples. In addition to the silver intrinsic absorption peak at approximately 400 nm, high transmission peaks are also found in the wavelength region of 600–690 nm for both the single- and double-layer silver dendritic samples. The two single-layer silver dendritic samples in Fig. 2d exhibit transmission peaks at 570 nm (green line) and 620 nm (black line), and the transmission peak of the double-layer silver dendritic sample in Fig. 2e occurs at approximately 620 nm. The size and pattern of the dendritic structures are varied through control of the electrochemical deposition voltage and time. As a result, the resonance frequencies of samples S1 and S2 are different. More sample responses in other visible wavelength ranges were prepared by fine-tuning the deposition conditions. Figure 2d and e also show the Polyvinyl Alcohol (PVA) antioxidation layer transmission curve (red line) in the visible light range with a transmission of approximately 90%, thereby showing that it can play a protective role in the sample without affecting the sample transmission. To improve the efficiency of the anomalous reflection and refraction, a multi-layer design can be used to enhance the resonance^30^. Compared with the single-layer silver dendritic sample, the overall transmission of the double-layer silver dendritic sample is reduced, whereas the transmission peak of the absolute height increases, and the transmittance difference between the resonant and non-resonant waveband increases. These results imply that the resonance of the multi-layer structure is enhanced significantly compared to the single-layer structure.

## GH shift measurement

The model suggested by Hess *et al*. indicated that an anomalous GH shift effect could occur in a metamaterial, thereby resulting in different wavelengths of light travelling at different points; this process is the physical basis of how to achieve slowing down of light^12^. We designed a test system to measure the anomalous GH shift effect in metamaterials using the methods proposed by Prajapati *et al*.^31^. The GH shift was measured using a 632.5 nm He-Ne laser. To determine the direction of GH shift of the silver dendritic metasurface, we also tested the direction of GH shift of the K9 crystal sample for comparison.

**Figure 3:**
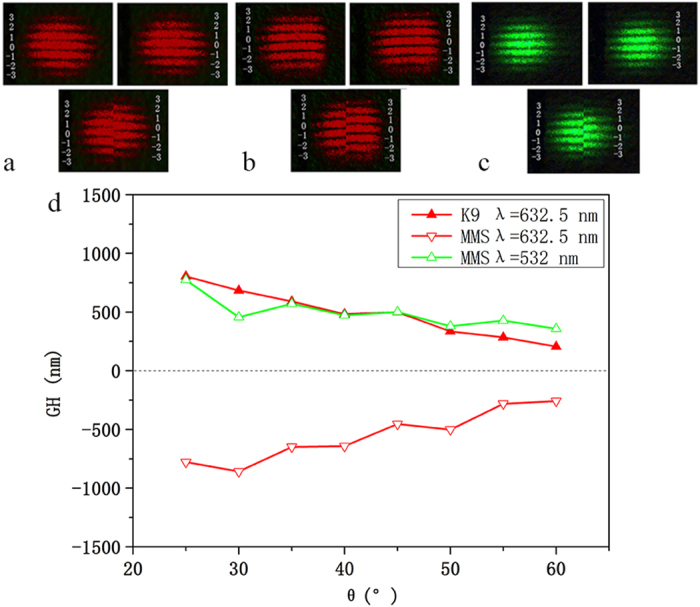
GH shift measurements for the K9 crystals and double-layer silver dendritic structure metasurfaces. The middle of the brightest interference fringe is labelled 0, and the upward and downward interference fringes are labelled ±1, ±2, and ±3, with the upward and downward cases represented by ‘+’ and ‘−’, respectively. The interference fringes were recorded when the analyser P2 was at 45° and 135°. The angle of incidence is 45°. The incident light wavelength is 632.5 nm. (**a**) GH shift of the K9 crystal sample. (**b**) GH shift of the silver-layer dendritic structure metasurface. (**c**) GH shift of the silver dendritic structure metasurface with an incident light wavelength of 532 nm. (**d**) GH shift values of different samples at different incident angles (approximately 25° to 60°).

Figure 3a and b show the interference fringe images of the K9 crystal and the silver dendritic metasurface sample, respectively, at a 45° incident angle. Comparing the two interference patterns, it is obvious that the GH shift of the silver dendritic metasurface sample is in the opposite direction to that of the K9 crystal sample. This observation reveals that the silver dendritic metasurface sample can exhibit an abnormal GH effect. In Fig. 3c, we illustrate the resulting GH shift when 532 nm light is incident on the silver dendritic metasurface sample. In this setting, the silver dendritic sample generates a normal GH shift similar to that of the K9 crystal sample. The above mentioned results indicate that the silver dendritic metasurface can achieve a positive GH shift at non-resonant wavelengths, whereas it can accomplish a negative GH shift in the resonance frequency range.

The results of further measurements of the GH shift at different incident angles are shown in Fig. 3d. The incident angle increases from 25° to 60°. For different wavelengths of incident light, the absolute value of the GH shift shows a general reduction with increasing incidence angle. Furthermore, the intensity and polarisation of the incident wave have no effect on the GH shift. The experimental result is obtained with a phase difference. The circularly polarised incident light is required in this experiment only. The thickness of the substrate also has no effect on GH shift because the GH shift occurs at the interface between the silver dendritic metasurface and air. For the incident light of 632.5 nm located within the sample resonant band, the GH shift value is negative. When the wavelength of the incident light is 532 nm (non-resonant frequency), the GH shift value is positive. These observations further confirm that the sample may have a negative GH shift because the incident light wavelength occurs at the resonance frequency of the sample. The GH shift at the interface between air and the dendritic metasurface is negative in the experimentally measured result, further illustrating that the dendritic metasurface is a material with a negative refractive index.

## Metasurface wedge waveguide

**Figure 4:**
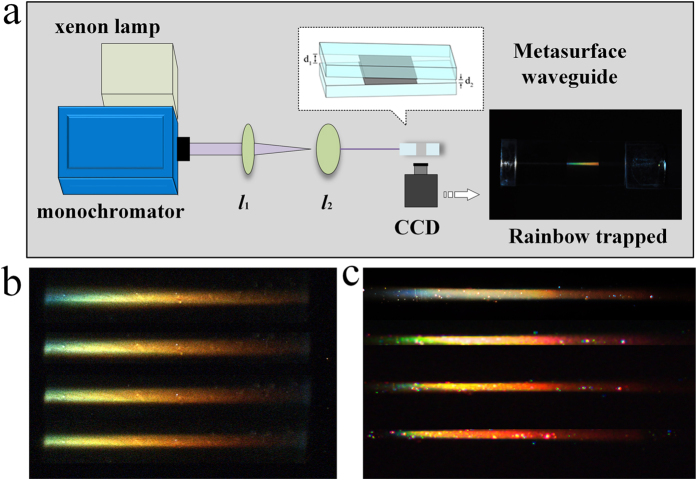
Rainbow trapping experiment. (**a**) A schematic of the experimental setup used to observe the rainbow trapping phenomenon. (**b**) Photographs of the rainbow trapping effect of a single-layer silver dendritic structure metasurface waveguide. (**c**) Photographs of the “rainbow trapping” effect of a double-layer silver dendritic structure metasurface waveguide.

Hess *et al*. proposed the use of an axially varying heterostructure to stop light at room temperature^12^. They inserted a metamaterial core with axially varying thickness between two different common materials and finally realised a rainbow trapping effect in the metamaterial core. This phenomenon was observed because the abnormal GH shift occurred on the interface of two media with opposite handedness. In contrast, we designed a wedge air waveguide structure constructed by two metasurfaces, as shown in Fig. 4a. Two samples with accordant resonant wavebands were combined to form the waveguide. The core of the silver dendritic metasurface waveguide is air, and the shell is a metasurface. As with the study by Hess *et al*. we obtained an abnormal GH shift on the interface of air and the dendritic metasurface. As a result, the constructed waveguide provides the physical basis to achieve rainbow trapping. The size of the leading port is *d*_1_, which is controlled by superimposing a thin gold foil layer by layer. The thickness of single-layer gold foil is 0.8 μm. In the experiments, the leading port was stationary, and the outlet of the waveguide was fixed to the vertical direction of the one-dimensional shift stage. Thus, the size of the rear port of the waveguide *d*_2_ can be tuned at the nanometre scale in real time.

Figure 4a shows the principle underlying the rainbow trapping experiments. We used a xenon light source (with a wavelength range of 250–1800 nm) combined with the monochromator to obtain a polychromatic light beam (zero-order diffraction grating light). The light beam passes through the wedge waveguide along the centreline. The rainbow trapping effect was observed at the top of the optical waveguide. Real-time observation of the incident light propagation phenomena in the waveguide was achieved using a Charge-coupled Device (CCD) camera. The inset of Fig. 4a shows the colour-separated stripes on the side surface of the waveguide. A significant distribution of colour can be observed from the left to the right along the incident light transmission path, corresponding to a colour change from a short wavelength purple light to the long wavelength red light. Although Hess *et al*. also predicted this phenomenon via theory, few experimental results on rainbow trapping obtained via a metasurface have been reported. In 2009, Zhao *et al*. experimentally demonstrated a trapped rainbow in tapered left-handed heterostructures at visible frequencies^14^ and found that the frequency components of the wave packet separated at positions with different guide thicknesses. In 2010, Smolyaninova *et al*. reported the experimental demonstration of two lasers transmitted through a structure of tapered optical nano-waveguide geometry and then separated into two circles^32^. In 2011, Gan *et al*. proposed the use of a metallic gradient chirp grating to experimentally observe a trapped rainbow via microscope images^33^,^34^. In this manner, the rainbow trapping phenomenon in the visible light spectrum provides a more obvious response across the full spectrum of the rainbow than the observations in previous experimental reports.

Theoretical studies have shown that under the adiabatic approximation condition, the change of waveguide thickness *d* along the waveguide length *l* is so small that the waveguide can be considered an approximately flat waveguide^31^. Thus, the rainbow trapping phenomenon can be realised. In our experiments, we adjusted the leading port *d*_1_ and the rear port *d*_2_ to meet the adiabatic approximation and successfully observed a clear rainbow trapping phenomenon. Figure 4b shows an image of a rainbow with single-layer silver dendritic sample. From the leading port to the rear port of the waveguide, the colours change from blue to yellow and finally to orange and red. The visible light distribution in the waveguide is incomplete. Some colours are missing. The results obtained using a double-layer silver dendritic metasurface waveguide are shown in Fig. 4c. Purple, blue, green, yellow, orange and other colours are all observed in the colour distribution, thereby forming a bright rainbow in the waveguide. We further observed the rainbow trapping effect of the wedge optical waveguide with various geometric parameters, wherein *d*_*1*_ = 2.4 μm and *d*_2_ is tuned in the range of 0–2 μm. The detailed results are shown in the supplementary material.

## Light power Measurement

We designed an experiment to test the incident power at the leading port of the waveguide and the output power at the rear port of the waveguide. A NOVAII handheld optical power tester with measurement accuracy of 0.001 nW was used to measure the light power. The experiments were conducted in an optical darkroom.

**Figure 5:**
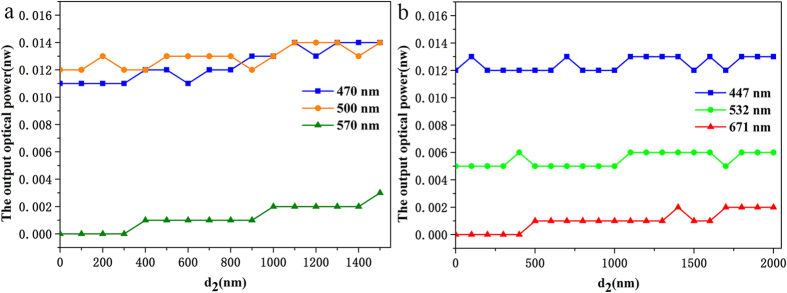
Evolution of the output optical power as the rear port *d*_2_ of the silver dendritic structure metasurface waveguide changes. (**a**) Results from a single-layer silver dendritic structure metasurface waveguide with different wavelengths of incident light. The optical input power is 0.065 nW, and *d*_*2*_ is from 0 to 1500 nm. (**b**) Results from a double-layer silver dendritic structure metasurface waveguide with different wavelengths of incident light. The optical input power is 0.470 nW, and *d*_2_ is within the range of 0 to approximately 2000 nm.

After repeated tests, we found that when the incident power was reduced to a certain extent, the output light power of the wedge waveguide could be reduced to 0.000 nW. However, with the same amount of energy incident on an ordinary glass waveguide, the output light power was approximately 0.023 nW. Figure 5 shows the test of impact of the outgoing light power with *d*_*2*_ size in single and double layers of the silver dendritic metasurface wedge waveguide. Figure 5a shows the output light power of the single-layer silver dendritic sample in the resonant and non-resonant wavelength band. When the incident light is 570 nm (resonant wavelength) with an input power of 0.065 nW, and *d*_2_ is from 0 nm to 300 nm, the output light power is 0.000 nW. For the incident light at a wavelength of 500 nm (non-resonant wavelength), the output light power is 0.012 nW. Further testing shows that the incident light wavelength is 470 nm (non-resonant wavelength), and the emitted light power is 0.011 nW. Figure 5b shows the measured results of the double-layer silver dendritic metasurface waveguide as *d*_2_ gradually increases from 0 nm to 2000 nm. When the incident light is 671 nm (resonance wavelength) with 0.472 nW optical power incident and *d*_2_ is from 0 nm to 400 nm, the emitted light power is 0.000 nW. This value is obtained because double-layer dendritic metasurfaces have higher efficiency than those comprising a single-layer. The results of single-layer and double-layer sample experiments show that when the incident light wavelength is in the resonant band and *d*_2_ is smaller than a certain value, the sample can exhibit the zero-flow phenomenon. The experimental result of an abnormal GH shift further proves that reverse power flow occurs in the waveguide. Therefore, the actual transmitted wave in the waveguide forms an effective thickness that is less than the physical thickness of the waveguide. When the effective thickness of the waveguide decreases to zero at a certain position, the wave completely stops and the power flow decreases to zero. This process provides a physical foundation to achieve the slowing down of light, i.e. the dendritic metasurface tapered waveguide meets the theoretical model which proposed by Hess *et al*.^12^.

We propose a dendritic cell cluster metasurface model that operates in the visible light spectral range. Numerical simulations show that when a light beam is incident on single- or double-layer dendritic metasurfaces, the reflection, refraction and incident waves are on the same side of the normal, in contrast to classical geometrical optics. This result implies that the silver dendritic metasurface is a material with a negative refractive index. The experimental results show that single- and double-layer silver dendritic metasurfaces can achieve negative GH shifts at the resonant frequencies of the metasurfaces. Further experiments confirm that at room temperature, a significant rainbow trapping effect can be observed in wedge waveguides composed of two silver dendritic metasurfaces, and the light power at the outlet of the waveguide is zero when the power of incident light is decreased into a certain amount; our findings thus suggest a new approach to slow down light. The silver dendritic metasurface can be prepared via a chemical method, and a large area can be prepared at low cost. The silver dendritic metasurface can also be readily extended to infrared and communications wavelengths. This result is a significant step in the development of optical information transmission and storage technology and has broad application prospects and development potential.

## Materials and Methods

### Simulation

Simulations were performed using CST Microwave Studio. In the simulations, we used the frequency domain solver. Periodic boundary conditions were used in the directions of the x- and y-axes, and an open boundary condition was used in the direction of the z-axis. The dendritic structural pattern is metal silver. The permittivity is described accurately by the Drude model with a plasma frequency of *ω*_*pl*_ = 1.37 × 10^16^ *s*^−1^ and a collision frequency of *ω*_*col*_ = 8.5 × 10^13^ *s*^−1^. The dielectric substrate is silicon with a permittivity of 11.9 F/m. The incident wave is the Transverse Electric(TE) mode. We studied the optical response behaviour of the silver dendritic metasurface in the visible light spectral range (400–750 nm).

### Preparation of the silver dendritic metasurface

The main electrochemical deposition process was conducted using an electrochemical workstation (Autolab, Metrohm, Herisau, Swiss Confederation). A silver plate with a purity of 99.9% and conductive glass were selected as the anode and cathode, respectively. The electrolytic solution was a mixed solution of silver nitrate (0.2 mg/ml) and PEG (polyethylene glycol) -20000 (0.24 mg/ml). The first silver dendritic structure was prepared under an applied voltage of 0.9 V DC over a deposition time of 90 s. To prevent the silver dendritic samples from being oxidised in air, we coated the surface with PVA film, which was prepared via the dip-coating method. The final result was the single-layer silver dendritic metasurface. In another case, after the completion of the first layer of the silver dendritic structure, a TiO_2_ dielectric layer was deposited by immersing the first layer of the silver dendritic structure in formulated butyl titanate anhydrous ethanol solution. The deposition voltage of the second layer of the silver dendritic structure was 1.8 V. The deposition time was 8 min. In this study, the ITO conductive glass surfaces (each with an area of 13 mm × 10 mm) with single-layer and double-layer silver dendritic structures were prepared. A Hitachi U-4100 spectrophotometer was used to measure the sample transmission within the wavelength region of approximately 300 nm to 900 nm. From the transmittance test of the sample, we found that the silver dendritic structure sample in the visible light spectral range had good resonant response. The pattern of each dendritic structure unit was indeed different in fabrication. However, the statistical distribution of the structure of the sample was definite. The resonance frequency can be changed by adjusting the external conditions, such as the electric field, the sedimentation time and the density of the electrolytic solution. In this manner, the resonance frequency of different samples can be made to be different. As a result, the presence of a rainbow and the field distribution can be altered for the different samples.

## Additional Information

**How to cite this article**: Fang, Z. H. *et al*. Slowing down light using a dendritic cell cluster metasurface waveguide. *Sci. Rep.* **6**, 37856; doi: 10.1038/srep37856 (2016).

**Publisher's note:** Springer Nature remains neutral with regard to jurisdictional claims in published maps and institutional affiliations.

## Supplementary Material

Supplementary Information

## Figures and Tables

**Figure 1:**
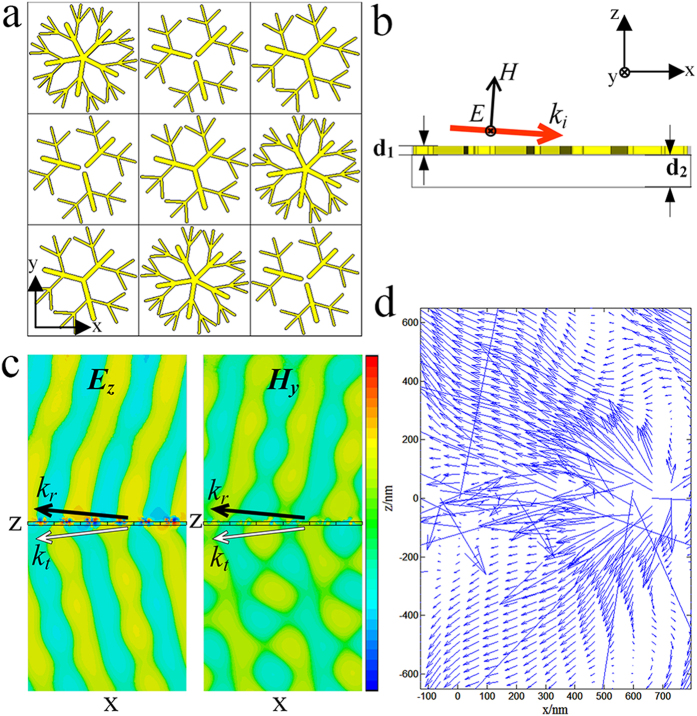
Silver dendritic metasurface structure, field distribution and power flux distribution. (**a**) A schematic structural view of the silver dendritic metasurface geometrical structure, which contains three types of units and is positioned in the x-y plane. An incident wave with an analogue frequency of 480 THz irradiates the metasurface at an 88° angle of incidence. (**b**) Side elevation of the dendritic metasurface. The thicknesses of the dendritic layer and the substrate are *d*_1_ = 12 nm and *d*_2_ = 40 nm, respectively. (**c**) A schematic of the field distribution; the position of the sample is shown in the dashed box. The wave direction is indicated by the black arrow. *k*_***r***_ and *k*_***t***_ are the reflection and the refraction wave vectors, respectively. The electrical field distribution shows that the reflected and refracted waves transmit in opposite manners. The same situation occurs in the magnetic field distribution. (**d**) A schematic of the power flux distribution. The direction of power flux is indicated by the blue arrow, and most of the energy is transmitted along the -x-axis; the energy transmits in the same direction as the reflected and refracted waves.

**Figure 2:**
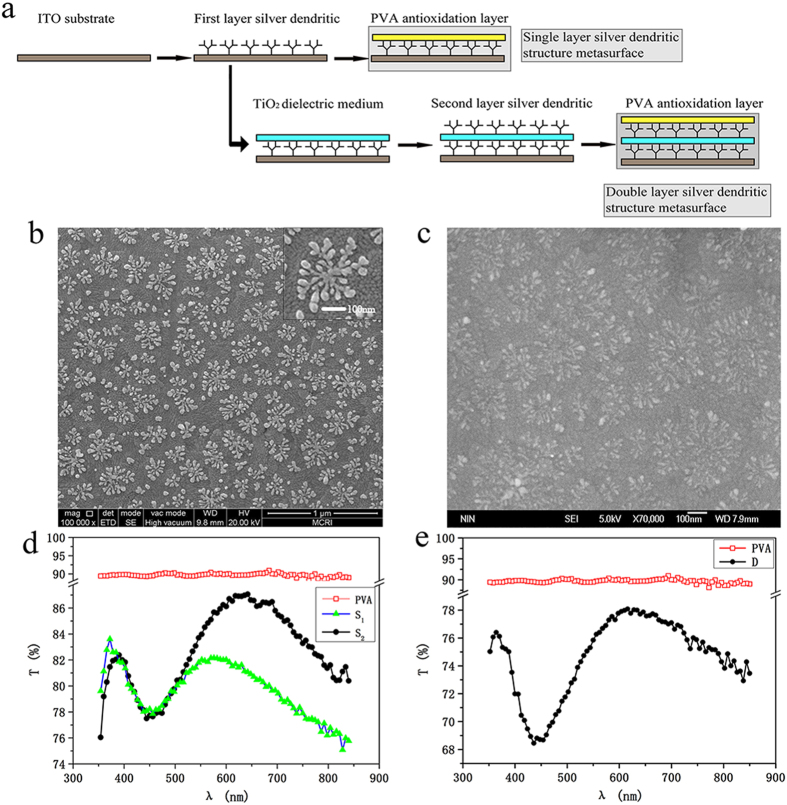
Preparation and characterisation of different silver dendritic metasurfaces. (**a**) A schematic of the preparation process of single-layer and double-layer silver dendritic metasurfaces. (**b**) Scanning Electronic Microscopy (SEM) photograph of a single-layer silver dendritic metasurface. The diameter of dendritic structural unit is approximately 230 nm. (**c**) SEM photographs of a double-layer silver dendritic metasurface. The diameter of dendritic structural unit in second layer is approximately 450 nm. (**d**) Transmission curves of a single-layer silver dendritic structure metasurface (S_1_ and S_2_) and a PVA antioxidation layer. (**e**) Transmission curves of a double-layer silver dendritic structure metasurface (D) and a PVA antioxidation layer.
