# Supplementary material for: Slowing down light using a dendritic cell cluster metasurface waveguide
Source: Sci Rep. 2016 Nov 25;6:37856. doi: 10.1038/srep37856 (PMC5122999; doi:10.1038/srep37856)
Supplement: Supplementary Information [file srep37856-s1.pdf]

Slowing down light using a dendritic cell cluster metasurface waveguide

Z. H. Fang, H. Chen, F. S. Yang, C. R. Luo & X. P. Zhao

Smart Materials Laboratory, Department of Applied Physics, Northwestern Polytechnical University, Xi'an 710129 P. R. China. Correspondence and requests for materials should be addressed to X.P.Z. (email: xpzhao@nwpu.edu.cn).

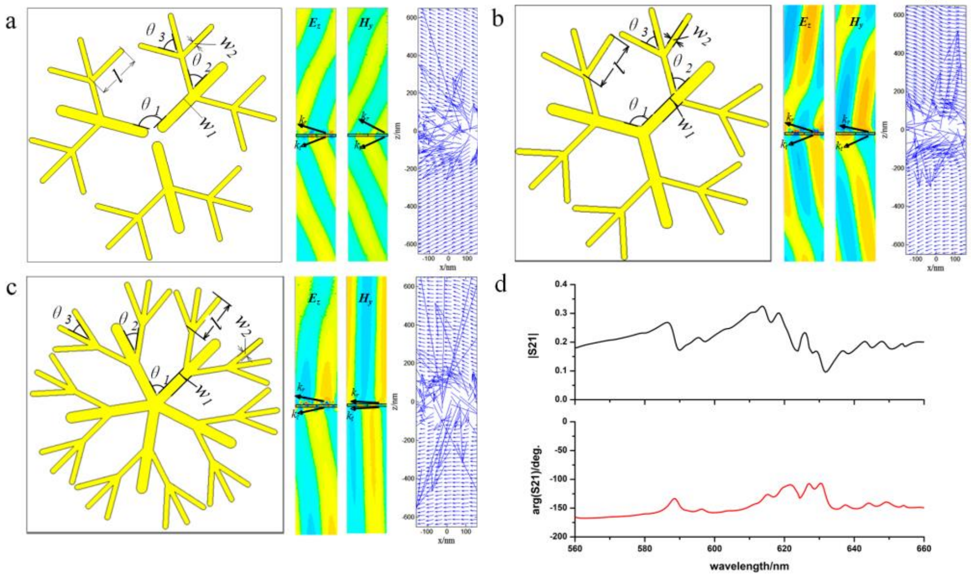

Fig. S1 Silver dendritic unit, field and energy flux distribution. Schematic of three geometrical structures of a silver dendritic unit, which is assembled with a rod in the *x*–*y* plane. The thicknesses of the silver dendritic layer and the substrate are 12 nm and 40 nm, respectively. Electrical and magnetic field distribution shows that the reflected and refracted waves are transmitted reversely. The direction of energy flux is shown by the blue arrow. **a** Rod length *l* is 52.31 nm. The width of narrower rod w_1 is 6.64 nm. The width of wider rod w_2 is 13.28 nm. The angle θ_1 between the neighbouring inner rods (the inside endpoints of which are not linked) is 120°. The angle θ_2 between the neighbouring middle rods is 60°, and the angle θ_3 between the neighbouring outer rods is also 60°. **b** Rod length *l* is 56.70 nm, rod width w_1 is 6.64 nm and rod width w_2 is 13.28 nm. The angle θ_1 is 120°, and the inside endpoints of inner rods are linked in this structure. The angles θ_2 and θ_3 are 60° and 48°, respectively. **c** Rod length *l* is 52.70 nm, rod width w_1 is 6.70 nm and rod width w_2 is 13.41 nm. The number of inner rods increases to five rods in this structure, and the angle θ_1 diminishes to 72°. The inside endpoints of inner rods are linked in this structure. The angles θ_2 and θ_3 are 36° and 30°, respectively. **d** Transmitted coefficient and phase curve of dendritic structure.

Simulation of a dendritic unit: A dendritic metasurface comprises different units.

The geometrical structure and simulation of silver dendritic units are illustrated in Fig.

S1. The thicknesses of the silver dendritic layer and the substrate are 12 nm and 40 nm, respectively, in Fig. S1 of the supplementary information. In addition, each of these three types of silver dendritic unit-cells is rotationally symmetrical. The dendritic unit is composed of a number of metal rods, and each rod has a fixed length of 52.31 nm. The unit morphology is shown in Fig. S1a. Three branches with an angle of 120° are found in between rods. The electrical and magnetic fields indicate that the reflection and transmission waves are transmitted reversely when the incident light yields a wavelength of 607 nm and reaches the metasurface with an angle of incident of i = 88. The energy flux and the wave vector are transmitted reversely in a similar transmission direction. If the length of the rod is elongated to 56.70 nm (Fig. S1b), the field and energy flux distribution of the unit exhibit the same behaviour. Conversely, the number of the branches increases to five and the angle in between rods changes to 72° if the length of the rods remains unchanged. Our simulation results are in accordance with the aforementioned conclusion (Fig. S1c). The transmitted coefficient and phase curve of dendritic structure are shown in Fig. S1d.

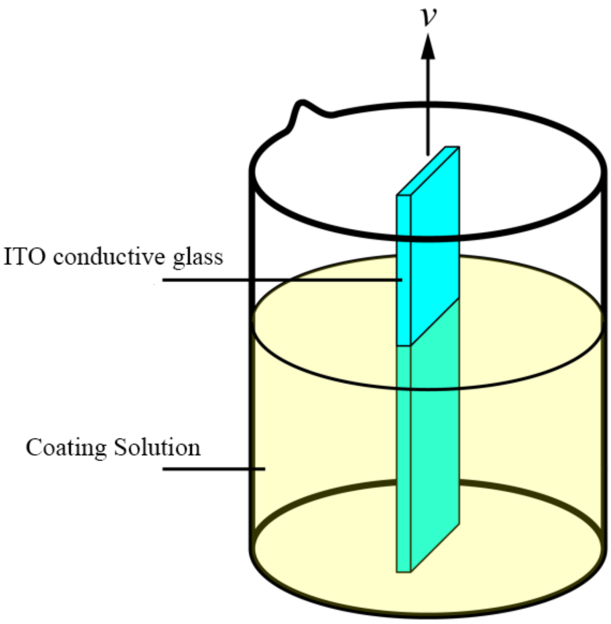

Fig S2 TiO2 film preparation technological process

Preparation of a double-layer silver dendritic metasurface sandwich of TiO_2: On the basis of the single-layer silver dendritic metasurface structure, we designed a double-layer silver dendritic metasurface sandwich. The selected dielectric layer of the sandwich was TiO_2. After the first layer of the silver dendritic metasurface was deposited, pulling coating was conducted to coat the TiO_2 dielectric layer. The substrate that deposited the first layer of the silver dendritic structure was fixed on a machine sample stage. Uniform immersion confected butyl titanate anhydrous ethanol. After infiltration was performed for 120 s, uniform pulling to the original height and the suspension was continuously baked for 15 min. The solution adhered to the substrate via water through the hydrolysis reaction of TiO_2 in air. Thus, a dense layer of TiO_2 films was formed.

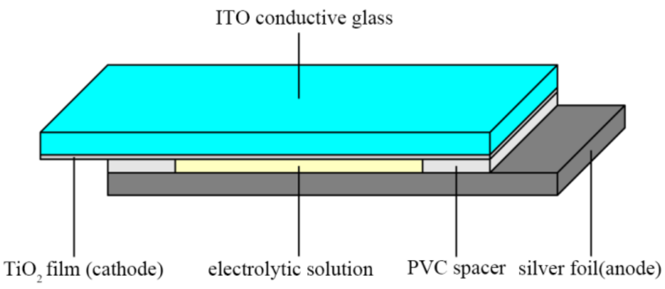

Fig S3 Schematic of electrochemical deposition process

The preparation of the second layer of the silver dendritic structure: We deposited a second layer of silver dendritic structures on TiO_2 films with a method similar to (1). In Fig. S3, a high-purity silver foil was placed horizontally on an autolab bench. The foil provided silver ions to deposit silver branches through the electrochemical deposition of an anode. Two uniformly thick PVC spacers were placed on the silver foil surface with a spacing of 1 cm. The substrates were deposited with a layer of silver branches and TiO_2 as a cathode upside down on the PVC spacer. These substances were added dropwise on the electrolyte side because the siphoning action of electrolytes was evenly distributed between the conductive glass and the silver foil. The autolab provided a constant voltage of 1.8 V. A cathode was added to the TiO_2 film because TiO_2 is a semiconductor, and its conductivity is smaller than that of ITO. As such, the deposition voltage and time should be altered. In this experiment, the deposition voltage and deposition time were appropriately adjusted. Thus, a second layer of silver dendritic structure was formed on the dielectric layer of the TiO_2 thin films. We obtained a composite structure containing dendritic silver/titanium dioxide film/dendritic silver. Under properly controlled conditions, these steps could be repeated to obtain bulk

optical metamaterials with three-dimensional multi-layer silver dendritic structures. In this study, optical metasurface with a 13 mm × 10 mm double-layer silver dendritic structure were prepared on the ITO conductive glass surface.

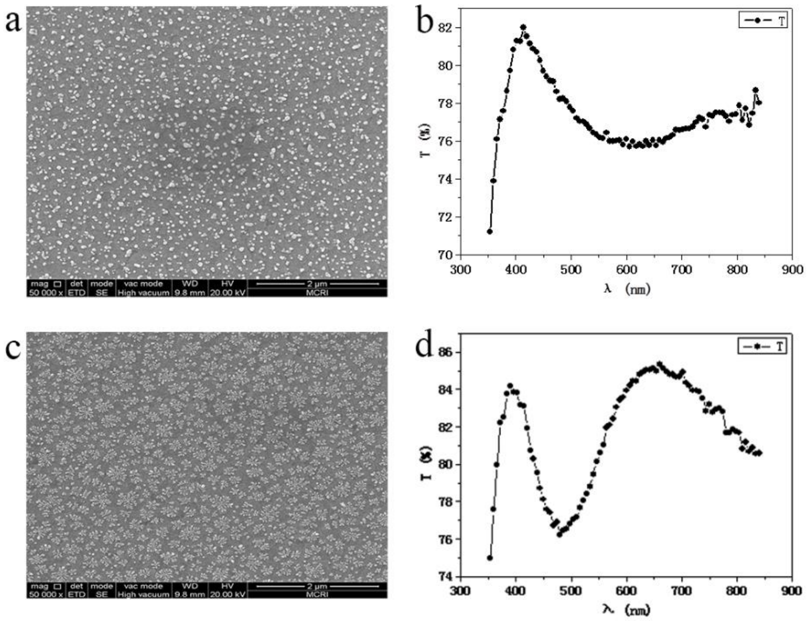

Fig. S4 Structure of the silver film and the silver dendritic units. a The SEM image of the silver membrane shows that silver granules are randomly distributed on the ITO surface. **b** The transmission curve of the silver film contains a silver intrinsic peak at approximately 400 nm. **c** The SEM image of the silver dendritic structure reveals silver dendrites distributed on the surface, and each unit measures 200–300 nm. **d** The transmission curve of the silver dendrites illustrates a specific transmission peak within the visible range.

Structure contrast of the silver film and the silver dendrites: We conducted a contrast experiment on the structures of the silver film and the silver dendrites to verify that the silver dendritic structure is the one that influences the performance of the sample, not the intrinsic characteristics of silver. A layer of silver granules was plated on the surface of two similar ITO conductive glass basements. The SEM image is presented in Fig. S4a. In the transmission image shown in Fig. S4b, only the intrinsic peak of silver is detected in the transmission map of the sample when a layer

of silver granules was plated on the basement. The transmittance of the sample reaches approximately 76% in the visible range and remains significantly unchanged. Fig. S4c illustrates the SEM image of the silver dendritic structure. Silver dendrites are distributed randomly on the basement, and each silver dendrite unit measures 200–300 nm. Fig. S4d shows the transmission map of the sample. A relatively high transmission peak, except the intrinsic peak of silver, exists in the visible range. This phenomenon indicates that the silver dendritic structure elicits a good resonance effect on visible light at this wavelength. Silver dendrite samples that respond to visible light at different wavelengths can be further obtained by controlling the preparation conditions.

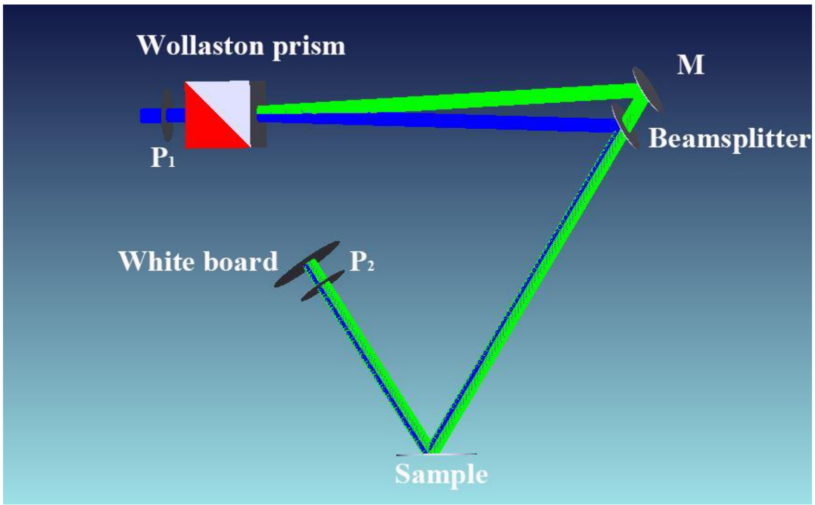

Fig. S5 Schematic of GH shift test settings. A circularly polarised red light at a wavelength of 632.5 nm was emitted when a He-Ne laser was used as a light source. P1 is a polariser, which transforms circularly polarised light into linearly polarised light. P2 is an analyser. Wollaston prism splits light into two beams, namely, *p*- and *s*-polarised light. They are placed vertically parallel to each other along the polarisation direction. *M* denotes the mirror, and BeamSplitter is the spectroscope. The white board receives interference patterns, and CCD records the experimental images.

GH displacement measurement: Since GH shift was discovered in 1947, researchers have worked with various models to explain and measure this parameter

accurately. Prajapati *et al.* developed an accurate measurement technique of GH shift using an interferometer and a Wollaston prism to separate incident polarised light into *s*- and *p*-polarised light. The light in the two parts of the sample surface interference fringes was obtained by analysing the calculated GH displacement. This method is simple and greatly improved compared with previous measurement methods. As such, we used this method to measure the anomalous GH shift of the silver dendritic metasurface in this study. The test settings are shown in the fig. S5. He-Ne was used as the light source, and the incident light was transformed into linearly polarised light through P1 with a polarisation angle of π/4. The linearly polarised light was then split into two beams of light, namely, *p*- and *s*-polarised light, by Wollaston prism. The *p*-polarised light was reflected to the back surface of the spectroscope by mirror *M*. A light column was reflected and the other column was transmitted. The *s*-polarised light directly travelled to the front surface of the spectroscope. A light column was reflected by the front surface and the other column was transmitted. On the front surface of the spectroscope, the reflection beam of **s** combines with the transmitted beam of **p**, and they travelled the same path until they reached the surface of the sample. The beam was also reflected by the surface of the sample. Afterward, the beam passed through the analyser, and the interference fringe appeared on the whiteboard. CCD simulated the phenomenon in real time. After the polarisation angles of the analyser were set at π/4 and 3π/4, that is, the angles between the analyser and the polariser were 0° and 90°, respectively, the interference image was recorded, transferred to a computer, and saved. The GH displacement of the sample was

calculated after the image was analysed.

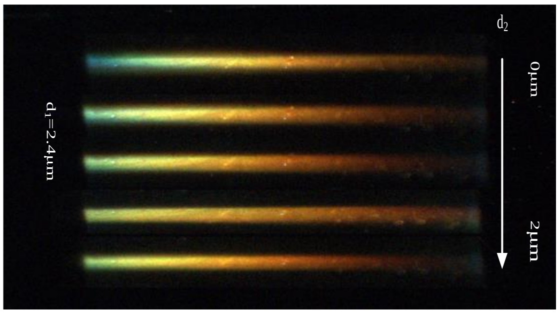

Fig. S6 Photographs of rainbow in single-layer dendritic waveguide.

Rainbow in different sizes of waveguide: Rainbow-trapped images could also different when the geometry of the optical waveguide of the wedge-shaped metasurface varied, where d_1 = 2.4 μm and d_2 is increased from 0μm to 2 μm. The results are shown in Fig. S6. A clear belt differs from the glass in front of the sample when light is over the surface, and evident colour distribution is not observed when d_2 is small, such as d_2 = 0, 200 nm. At d_2 of 400 and 600 nm, a bright coloured light ranging from purple to red appears. This finding indicates that the different colours of light stay in the different thicknesses of the waveguide; thus, spatial spectral separation, that is, rainbow-trapping effect. As d_2 increases, the rainbow moves to the right. Some light wavelengths 'stay' in a position corresponding to a specific thickness of the core layer. When d_2 is 800 nm, a bright 'rainbow' can be observed in the wedge metasurface waveguide. At a high d_2, such as d_2 = 1800 nm, the rainbow in the waveguide of the metasurface disappeared, but a dark grey light band appeared. The waveguide thickness does not satisfy the conditions of adiabatic approximation. If d_2 is greater than the critical thickness, then the visible light unlikely 'stays' in the waveguide. Most of the light is transmitted out of the rear port. As a result, a rainbow

is not formed.
